# Rational Design of Pore Parameters in Monodisperse Porous Poly(glycidyl methacrylate-*co*-ethylene glycol dimethacrylate) Particles Based on Response Surface Methodology

**DOI:** 10.3390/polym14030382

**Published:** 2022-01-19

**Authors:** Julia C. Steinbach, Fabio Fait, Stefanie Wagner, Alexandra Wagner, Marc Brecht, Hermann A. Mayer, Andreas Kandelbauer

**Affiliations:** 1 Process Analysis & Technology, Reutlingen Research Institute, Reutlingen University, Alteburgstraße 150, 72762 Reutlingen, Germany; Julia.Steinbach@Reutlingen-University.de (J.C.S.); Fabio.Fait@Reutlingen-University.de (F.F.); Alexandra.Wagner@Reutlingen-University.de (A.W.); Marc.Brecht@Reutlingen-University.de (M.B.); 2 Institute of Inorganic Chemistry, University of Tübingen, Auf der Morgenstelle 18, 72076 Tübingen, Germany; stefaniewagnersb@googlemail.com (S.W.); hermann.mayer@uni-tuebingen.de (H.A.M.); 3 Institute of Physical and Theoretical Chemistry, University of Tübingen, Auf der Morgenstelle 18, 72076 Tübingen, Germany; 4 Department of Material Sciences and Process Engineering (MAP), Institute of Wood Technology and Renewable Materials, University of Natural Resources and Life Sciences, Gregor-Mendel-Straße 33, 1180 Vienna, Austria

**Keywords:** porous microspheres, design of experiment, seed swelling polymerization, p(GMA-*co*-EDMA), particles, monodisperse, pores, morphology, process optimization

## Abstract

Monodisperse porous poly(glycidyl methacrylate-*co*–ethylene glycol dimethacrylate) particles are widely applied in different fields, as their pore properties can be influenced and functionalization of the epoxy group is versatile. However, the adjustment of parameters which control morphology and pore properties such as pore volume, pore size and specific surface area is scarcely available. In this work, the effects of the process factors monomer:porogen ratio, GMA:EDMA ratio and composition of the porogen mixture on the response variables pore volume, pore size and specific surface area are investigated using a face centered central composite design. Non-linear effects of the process factors and second order interaction effects between them were identified. Despite the complex interplay of the process factors, targeted control of the pore properties was possible. For each response a response surface model was derived with high predictive power (all R^2^_predicted_ > 0.85). All models were tested by four external validation experiments and their validity and predictive power was demonstrated.

## 1. Introduction

Porous polymer particles have attracted considerable interest in various applications ranging from clinical diagnostics [1], immobilization support for biocatalysts [2], separation processes like chromatography [3,4,5,6], ion exchange phases [7] to applications as adsorption materials [8,9] or as hard-templates [10,11,12] for inorganic materials with defined structures. In particular, porous poly(glycidyl methacrylate-*co*-ethylene glycol dimethacrylate) (p(GMA-*co*-EDMA)) has been widely used, as the epoxy ring of GMA can be derivatized in various ways by ring opening reactions [2,13,14,15]. This allows application-specific functionalization and makes the p(GMA-*co*-EDMA) a versatile platform polymer. p(GMA-*co*-EDMA) particles have been used as chromatographic column material [4,16], as carriers for biocatalysts [17], for solid phase extraction [8] or as hard template for porous silica particles with defined pore structure [11].

For many applications involving particles, a small size distribution is advantageous. Monodispersity in these porous microspheres can be achieved through seeded swelling polymerization [18,19]. This approach allows one not only to influence the dispersity and size of the microspheres, but also to tailor the surface properties [20]. Thereby the increase in size is mainly dependent of the ratio of the organic phase to the amount of seed particles [21,22]. It has been reported that the specific surface area, porosity and pore volume depend on the composition of the organic phase of seeded swelling polymerization [21,23,24,25,26,27,28]. The organic phase usually consists of the monomers, here EDMA as crosslinker and GMA as monomer with a functional epoxide ring, and the porogen mixture, which are inert solvents, here cyclohexanol and toluene.

However, due to the high complexity of the system and the dependency on the different solubilities of the components, previous studies have been rather confined to semi quantitative statements from considerations of isolated process factors (“one-factor-at-a-time”, OFAT), which do not allow a specific control of the synthesis and the pore and surface properties. In contrast to the common OFAT approach, in response surface methodology (RSM) multiple process factors are simultaneously and systematically varied and their effects are analyzed using a statistical approach. RSM provides not only insight in the effect strengths of single process factors but also allows to detect the presence of non-linear behavior and synergistic interactions between multiple process factors. This provides a detailed causal model of the studied process. [29,30,31] Currently, to the best of our knowledge, there is no predictive causal model available that is actually based on the numerical effects of the process factors on the morphological properties of porous p(GMA-*co*-EDMA) particles and thus allow for demand-driven control of particle properties.

The present study aims at demonstrating a valid and robust model allowing for tailored modification of pore parameters in porous p(GMA-*co*-EDMA) particles using RSM. For this purpose, the statistical significance and effect strengths of the process factors involved in the synthesis (a) monomer:porogen ratio, (b) GMA:EDMA ratio and (c) composition of the porogen mixture (toluene:cyclohexanol) were determined with respect to the responses pore volume, pore size and specific surface area using a face centered central composite experimental design. The pore properties were analyzed using inverse size exclusion chromatography (iSEC). To enable further application-specific functionalization, the conversion of the epoxide groups in the particles was studied by FTIR in combination with multivariate calibration by partial least squares regression (PLSR). Additional particles were prepared using four sets of factor level combinations as external validation experiments and characterized to verify the robustness and validity of the response surface models (RSM) established. The RSM approach has offered a better understanding of the seeded swelling polymerization for porous p(GMA-*co*-EDMA) and has provided valid, predictive models for the pore volume, pore size and specific surface area, which allow targeted adjustment of these quantities.

## 2. Materials and Methods

### 2.1. Chemicals

Styrene (99%), and ethylene glycole dimethacrylate (EDMA, 98%) were purchased from Fisher Scientific GmbH (Schwerte, Germany). Polyvinylpyrrolidone K30 (PVP), benzoyl peroxide (BPO, 75%), dibutyl phthalate (DBP, 99%) and glycidyl methacrylate (GMA, ≥97%) were purchased from Sigma-Aldrich Chemie GmbH (Taufkirchen, Germany). Polyvinyl alcohol (PVA, hydrolyzed 86–89%) was purchased from abcr GmbH (Karlsruhe, Germany). Sodium dodecyl sulfate (SDS, ≥99%) was purchased from Carl Roth GmbH + Co. KG (Karlsruhe, Germany). Ethanol 96% was purchased from VWR Chemicals (Darmstadt, Germany). All chemicals were used as delivered.

For the iSEC analysis tetrahydrofuran 99.9% was purchased from Carl Roth GmbH + Co. KG (Karlsruhe, Germany) and the polystyrene (PS) standards were purchased from PSS Polymer Standards Service GmbH (Mainz, Germany).

### 2.2. Instrumentation

An Ultra-Turrax T50 (IKA^®^-Werke GmbH & CO. KG, Staufen, Germany) was used for homogenization of the prepared emulsions.

iSEC measurements were carried out using an Agilent 1100 series system (Agilent Technologies, Waldbronn, Germany) equipped with a quaternary pump with degasser, an auto sampling system, 6-port valve for column selection and a DAD detector (254 nm). Instrument control, data acquisition and automated data analysis was performed by the Chem-Station software (B.04.03, Agilent Technologies, Waldbronn, Germany).

Spectral characterization was performed using a Frontier 2 FTIR (PerkinElmer LAS GmbH, Rodgau, Germany) with an attenuated total reflection accessory. The resolution was 2 cm^−1^ with 4 scans. For data processing the spectral region was reduced to the region from 1750 to 700 cm^−1^ which carried the highest amount of relevant information. The spectra were smoothed using a Savitzky-Golay filter and were treated with standard normal variate transformation, to remove multiplicative scattering interferences [32,33].

A partial least square regression model (PLSR, kernel algorithm) was applied for multivariate calibration of the relative GMA ratio. A total of 155 spectra from particles Std1–19 were used for modelling. From the 24 spectra of particles Val1–4 the predictive power of the model was verified. The processing software Unscrambler-X (CAMO Software AS, version 10.5.1, Oslo, Norway) was employed for spectral preprocessing, spectral analysis and multivariate data analysis.

### 2.3. Seeded Suspension Polymerization of Porous p(GMA-co-EDMA) Particles

The porous p(GMA-*co*-EDMA) particles were synthesized by seeded suspension polymerization using toluene and cyclohexanol as porogens. The polystyrene (PS) seeds were prepared according to the procedure provided in the Appendix A. 0.3 g PS seeds and 5 mL of a 2.0 g∙L^−1^ SDS solution were sonicated for 10 min. An emulsion containing 2.0 mL of the activator DBP and 150 mL of SDS (2.53 g∙L^−1^) were homogenized for 10 min at 5000 rpm and added to the seed particle suspension. The mixture was stirred for 24 h at 200 rpm at room temperature. Thereafter an emulsion (10 min, 5000 rpm) containing 150 mL of SDS (3.33 g∙L^−1^), initiator BPO (0.4 g), porogens and acrylate monomers was added. The relative amount of added monomers (GMA:EMDA) and porogens (toluene:cyclohexanol) was systematically varied according to the experimental design (Table 1). The absolute volume of added monomers and porogens was 30 mL for each synthesis.

To allow for complete swelling, the system was stirred at 200 rpm for another 24 h. 150 mL of a 23.3 g∙L^−1^ PVA solution was added to the mixture as stabilizer. The reaction mixture was heated to 70 °C and stirred at 200 rpm for 24 h for polymerization. After polymerization the porous particles were filtered off and washed three times with ethanol and three times with water. The porous copolymer particles were then dried prior to analysis.

### 2.4. Experimental Design

A face-centered central composite experimental design (FCD) was set up, allowing the mathematical description of non-linear terms [34,35]. A total of 19 particle batches were synthesized with five center point (CP) replications to determine reproducibility and system variance.

The effects of three factors were investigated by systematic variation according to an FCD response surface design, with the low, intermediate and high factor level settings given in Table 1. As factors the ratio of monomer (methacrylates) to porogens (A: monomer:porogen vol%), the ratio of monomer with the functional group (GMA) to the crosslinker (EDMA) (B: GMA:EDMA vol%) and the ratio of the porogens toluene to cyclohexanol (C: toluene:cyclohexanol vol%) were varied.

The effects were analyzed using analysis of variance (ANOVA). The significance level was α = 0.05, meaning that a model or model term was considered statistically significant if its *p*-value was *p* ≤ 0.05.

For model validation four additional syntheses within the design space were conducted. The validation experiments Val1–Val4 were selected as a series with increasing GMA content moving through the experimental space along one factor axis, see (Table 2). This would correspond to a one-factor-at-a-time experiment. The run order is the randomized order in which the experiments were actually performed to compensate for systematic errors.

### 2.5. Pore Characterization Using Inverse Size Exclusion Chromatography (iSEC)

The particles were packed into stainless steel columns (4.6 × 250 mm) using the slurry method with ethanol/water (50:50 vol%:vol%). For characterization of the pore properties the columns were analyzed by iSEC. A set of 19 polystyrene (PS) standards from *M_w_* = 162 Da–2.5 mDa with a molar-mass dispersity of < 1.1 were applied. 10 µL of each 2.5 g∙L^−1^ PS in THF were separately injected (10 µL) three times. The lowest molecular weight standard was used as a t_0_ marker. All standards were measured with THF as eluent and a flow rate of 0.25 mL∙min^–1^.

The pore parameters were analyzed in accordance to the method of Halász et al. (1978) [36]. The pore volume *V_p_* is calculated using Equation (1):

(1) $$V_{p}=V_{0}-V_{i}\;$$

as the difference between the dead volume *V*_0_ (=elution volume of t_0_ marker) and *V_i_* the interstitial volume (=elution volume of totally permeating volume).

The distribution coefficient *K_SEC_* is dependent on the pore size of the particles. It can be derived from the experimentally determined elution volume *V_e_*, the interstitial volume *V_i_* and the elution volume of the totally permeating solute *V_t_*, see Equations (2) and (3):

(2) $$V_{e}=V_{i}+V_{p}\times K_{SEC}\;$$

(3) $$K_{SEC}=\frac{V_{e}-V_{i}}{V_{p}}$$

According to Halász et al. the exclusion values (pore size) *Φ* in Å of linear PS in a good solvent can be calculated based on the molecular weights *M_w_* (Da) of the PS standards as follows:

(4) $$\Phi ≅0.62\;M_{w}{}^{0.59}$$

The mean pore size *Φ*_50_ is determined from the lognormal distribution plot of *K_SEC_* as function of log(*Φ*) the at *K_SEC_* = 50%.

From the pore volume *V_p_* (mL) and the mean pore size *Φ*_50_ (Å) the specific surface area *S* can be derived according to Equation (5):

(5) $$S=\frac{4000\times V_{p}}{\Phi_{50}}$$

### 2.6. Scanning Electron Microscopy Images (SEM)

For the evaluation of morphology, particle size and dispersity SEM images were acquired using a Hitachi SU8030 (Hitachi High-Tech Europe GmbH, Krefeld, Germany). The size and dispersity were assessed semi-automatically from the SEM images using a self-written MATLAB script. 400–500 particles were measured (min. 374), except for Std7 were no sufficient number of intact particles could be evaluated. The median particle diameter is given as particle size in µm. The dispersity is given by the d_90_/d_10_ value which indicates the width of the particle size distribution. Here d_90_ is the value below which 90% of the distribution lies, with d_10_ corresponding to 10% of the distribution. A d_90_/d_10_ value smaller than 1.4 is considered as a monodisperse distribution.

## 3. Results and Discussion

For systematic evaluation of the synthesis factors which influence the particle size, pore volume, pore size, specific surface area and morphology of the porous particles, approach of RSM was applied. Compared to single-factor variation-based experiments, RSM not only offers a more time- and resource-efficient approach, but also allows the determination of non-linear effects and interactions of the parameters [37].

Factor level settings of the process factors A: monomer:porogen ratio, B: GMA:EDMA ratio and the porogen composition C: toluene:cyclohexanol were systematically varied according to the FCD design given in Table 2. All p(GMA-*co*-EDMA) particles Std1–19 (Table 2) were synthesized using a seeded swelling polymerization process. The employed seed PS particles were 1.95 µm in size and had a narrow size distribution of d_90_/d_10_ = 1.09 (span: 0.09). They were all from the same batch. Std1 and Std3 were defined as outliers due to their untypically large residuals and increased lack of fit. These two experiments were excluded from further analysis.

### 3.1. Particle Size and Dispersity

**Figure 1:**
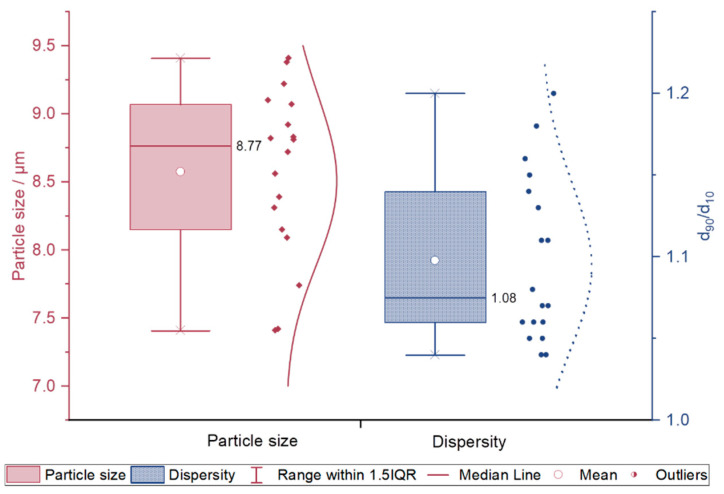
Box-Whisker-Plot with lognormal distribution curve of single values for particle sizes (left hand, red) and d_90_/d_10_ (right hand, blue) of standard order 1–19. Box displays 1. and 3. quartile, whiskers display 1. and 3. quantile + 1.5∙interquartile range. x = minimum and maximum values.

The p(GMA-*co*-EDMA) particle sizes varied between 7.41–9.38 µm and particles of all batches showed a monodisperse distribution with a d_90_/d_10_ range between 1.04 and 1.20 (CV% max. 15.8%), see Figure 1. No statistically significant effects of the factors on the dispersity were found. The median d_90_/d_10_ is 1.08. This is in accordance with the finding that the dispersity of the seed particle is crucial for the dispersity of the product [38].

For the final particle size, a statistically significant effect (*p* = 0.0004) of the porogen composition is found. The model equation in terms of coded factors of the effect onto the particle size is given in Equation (6):

Particle size/µm = 8.69 + 0.5521 C(6)

The higher the ratio of toluene:cyclohexanol/vol% larger particles are obtained (*p* = 0.0004). The model quality parameters are R^2^ = 0.6070 and R^2^_predicted_ = 0.4942.

### 3.2. Morphology

**Figure 2:**
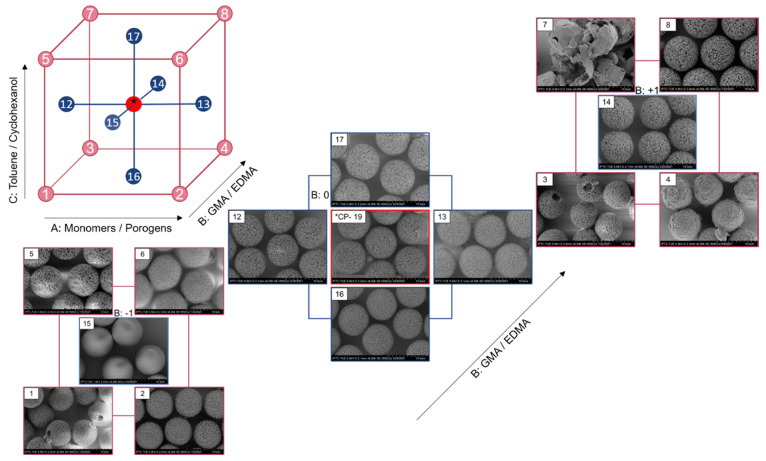
SEM images with 10,000 magnification of each particle batch numbered by standard order. The design cube is divided according to the level of factor B: GMA:EDMA ratio. Factorial points are numbered according to the standard order given in Table 2 and are displayed burgundy, central point is displayed red and axial points are displayed blue.

A clear change in morphological characteristics is observed with the systematic variation factor level settings, (Figure 2). Increasing the monomer ratio of the organic phase, generates less porous particles. This effect is especially pronounced at high levels of GMA content of the monomers. The toluene:cyclohexanol ratio strongly affects the surface roughness. While a high content of toluene generates a smooth particle surface, high cyclohexanol content leads to a rough dimpled appearance. The roughness and surface irregularities are intensified with increasing GMA ratio in the monomer composition.

Interestingly, Std1 and Std3 show a hole at one side of the particle, which is approximately 2 µm in diameter. Both batches were synthesized with a high content of cyclohexanol as the porogen (70 vol% of organic phase). Similar behavior was found earlier when high amounts of cyclohexanol were used [19,39]. This finding can be related to the solubility of the seed particle PS in the overall composition of the organic phase. The suitability of solvents can be approximated using the relative energy difference (RED), which is the ratio of the distance in Hansen space *R_a_* to the radius of the interaction sphere in the Hansen space *R*_0_ (here for PS). Good solvents show RED values below 1, solvents around 1 are found to only partly dissolve the solute (or swell it), while RED values higher are non-solvents. [40] Cyclohexanol is the least suitable solvent for PS used in the experiments and shows a RED of 0.96 (GMA 0.82, toluene 0.65, DBP 0.59). While the seed particle is soluble in the organic phase still containing the monomers, with progressing polymerization the solubility decreases until the solubility limit is exceeded and the seed particle is reformed. Therefore, no polymerization of methacrylate takes place in this area, leaving a hole after the PS is detached during purification. In contrast to the syntheses shown here, the seed swelling polymerization of p(GMA-*co*-EDMA) using 1-hexanol as porogen results in polystyrene caps persisting on the particle surface instead of holes [41]. A dependence on the solubilities is assumed. This could also be the reason for the higher studentized residuals, for the analysis see Table 2.

With the reaction conditions Std7 with a high porogen content consisting of 100 vol% toluene and a high GMA ratio of monomers, no stable particles were obtained. From SEM pictures the particles look like empty shells or skins with a smooth outer layer (Figure 2). This phenomenon could result from low solubility of toluene in water, leading to a displacement of monomers at the interface with the continuous aqueous phase to minimize surface tension. Thus, a polymeric skin is formed during polymerization around the toluene which is present in and accumulated on the inside of the particle [20,42].

Particles obtained with factor settings according to Std4 exhibited no visible porosity, but rather outwardly curved dimple structures (Figure 2). This effect results from high monomer content with small ratio of GMA and the usage of pure cyclohexanol as porogen. Due to the lack in porosity no iSEC analysis could be performed with these particles.

### 3.3. Multivariate Calibration Model for Epoxy Functionalization

**Figure 3:**
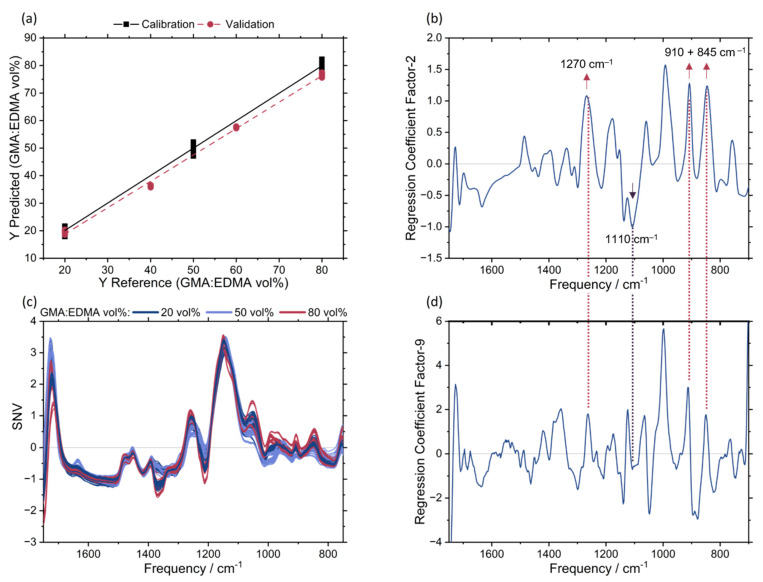
PLSR with nine factors for GMA:EDMA ratio based on FTIR spectra. (**a**) shows the predicted vs. reference of GMA:EDMA ratio for calibration (■) and validation (●). The SNV pre-treated spectra of GMA:EDMA ratio of 20 vol% (dark blue), 50 vol% (light blue) and 80 vol% (red) are displayed in (**c**). (**b**,**d**) show the regression coefficients for factor two (highest explained variance) and factor nine (model regression coefficient) and influential frequencies are highlighted.

**Figure A1:**
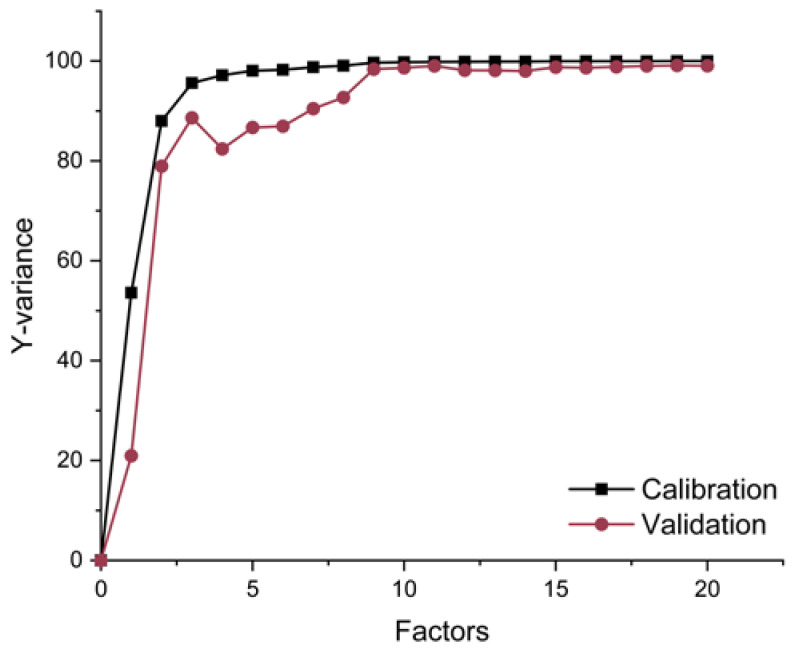
Plot of explained variance for the PLSR depending on the factors used to predict epoxy functionalization for calibration (■) and validation (●).

With the epoxy group as functional group the p(GMA-*co*-EDMA) particles can easily be converted to a desired functionalization. For multivariate calibration a PLSR model based on nine factors was calculated from the spectra of Std1–19 (Figure 3). To test the predictive power of the model, the GMA content of validation experiments Val1–4 was predicted (Table 3), based on the corresponding SNV pre-treated spectra (Figure 3c). The explained variance plot is given in Appendix B, Figure A1.

With an R^2^_calibration_ = 0.99 and R^2^_predicted_ = 0.98 the model quality is very good (Figure 3a). The root-mean-square-error of calibration is 1.2 vol%, and that of prediction is 2.9 vol% GMA. The model allows an accurate determination of the GMA:EDMA monomer ratio (relative deviation predicted/actual < 5%) used in the synthesis (Table 3). The highest explained variances lie on factors 1 and 2 (88%, Figure 3b). However, due to the complex interplay between the process factors used in the system [33], nine regression coefficients are required to build a robust model with high predictive power. From the regression coefficients, the relevant absorbance signals carrying the distinguishing information can be identified. The most important frequencies are indicated in Figure 3b.

For increasing EDMA ratio (and vice versa decreasing GMA content) the symmetric C-O-C stretching vibration at 1110 cm^−1^ [43] is increasing (Figure 3). The characteristic frequencies increasing with GMA ratio are mainly related to vibrations of the epoxide group, with the symmetric and asymmetric ring vibration of the epoxide ring at 845 cm^−1^ [43] and 910 cm^−1^ [43], respectively. The C-O stretching vibration of the epoxide ring at 1270 cm^−1^ [43] also responds to changes in GMA ratio. This shows that the monomers are converted proportional to the mixture components and epoxide groups are present in the respective amounts in the final product. Hence, the degree of functionalization can be controlled.

### 3.4. Pore Volume

The effects of the process factors on the pore volume were analyzed by ANOVA are given in Table 4.

A statistically significant model (*p* < 0.0001) describing the pore volume in dependence of four effects was obtained. The effect of factors monomer:porogen ratio (p < 0.0001), toluene:cyclohexanol ratio (*p* < 0.0001) and the interaction effect between GMA:EDMA ratio and toluene:cyclohexanol ratio (*p* = 0.0049) are statistically significant. The linear term B (GMA:EDMA ratio) is only added to comply with model hierarchy [44]; since B is involved in an interaction effect it must be included in the model although by itself B was no statistically significant effect term. The lack of fit is non-significant with *p* > 0.05. The strengths of the effects are given as coded equation in Equation (7):

V_p_/mL∙g^−1^ = 0.2903 − 0.1984 A − 0.0249 B + 0.1366 C + 0.0749 BC (7)

Equation (7) shows a linear negative effect of A: monomer:porogen ratio on the pore volume and is also the strongest effect. As the amount of monomer increases or the amount of porogen on the organic phase decreases during swelling, the polymer network becomes denser, less space is occupied by inert porogen, and thus the pore volume is reduced [39,45].

However, Figure 3 and Equation (7) show that not only the monomer:porogen ratio influences the pore volume, but also the composition of the porogen mixture and its synergistic interaction with the GMA:EDMA ratio. The porogen composition was examined in a range of 0/100 *v*/*v* ratio of toluene:cyclohexanol to a ratio of 100/0 *v*/*v* of toluene:cyclohexanol.

Depending on the suitability of the solvent for the polymer either χ-induced or ν-induced syneresis takes place, corresponding to a phase separation before or after the gel point, respectively [42]. Good solvents lead to smaller pores, smaller pore volumes and larger surface areas through ν-induces syneresis. [42] Nonsolvent or even linear polymeric porogens, like the PS from the seed particle, lead to larger pores with higher pore volume but reduced specific surface area through χ-induced syneresis [4]. According to the Hildebrandt solubilities of the p(GMA-*co*-EDMA) polymer with 24.0 Mpa^1/2^ [46], toluene with 18.2 MPa^1/2^ [40] and cyclohexanol with 22.4 MPa^1/2^ [40], cyclohexanol is a better suited solvent for the polymer and should therefore result in smaller pores and smaller pore volumes. Since the amount of PS seed is constant, its influence as linear polymeric porogen is assumed to be constant for all conducted syntheses.

The factor C: toluene vol% shows a generally positive effect on the pore volume. With increasing amount of toluene in the porogen composition, the pore volume increases as well. This is supported by the Hildebrand solubilities.

**Figure 4:**
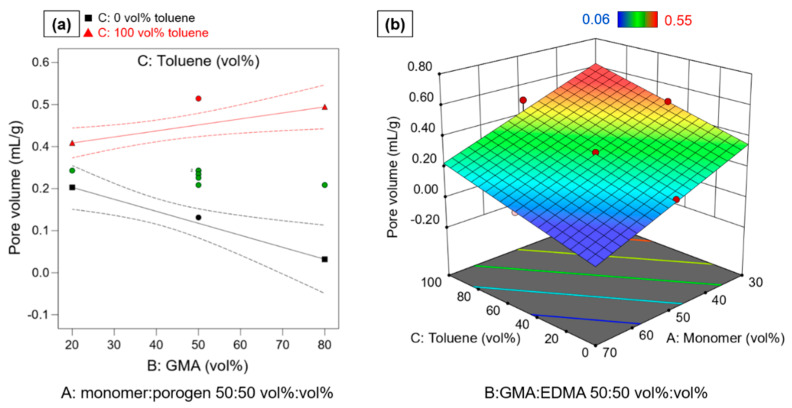
(**a**) Interaction plot of BC GMA:EDMA*toluene:cyclohexanol at medium level of factor A. Red triangles indicate measured pore volume at high toluene content. Black squares indicate measure particle volume at 0 vol% toluene (100 vol% cyclohexanol). Green circles indicate measured particle volume at medium toluene level. Dashed lines indicate 95% confidential intervals. (**b**) Shows response surface of pore volume in dependence of factors A and C. Blue areas correspond to low pore volumes, red areas correspond to high pore volume.

However, Figure 4a shows that this relationship is not linear, since the interaction term BC between GMA:EDMA ratio and porogen composition has a high significant (*p* = 0.0066). While particles which have a low GMA content, show similar pore volumes for high and low toluene ratio in the porogen mixture, a difference for particles with high GMA content is visible. With increasing GMA content lower pore volumes are obtained with low toluene ratio whereas high pore volumes are accessible with a high toluene ratio (Figure 4b). The interaction BC could therefore be caused by changing solubilities depending on the porogen and monomer mixture, indicating a better solubility of GMA in cyclohexanol. The relationships of the factors on the pore volume are very complex, due to the influence of the solubilities. Nevertheless, the model shows a very good correlation with R^2^ = 0.9688 and R^2^_predicted_ = 0.9012.

### 3.5. Pore Size

The effects of the variations in process factor levels on pore size were also found to be highly statistically significant (*p* < 0.0001). The pore size is dependent on factor A: monomer:porogen ratio, factor B: GMA:EDMA ratio, and factor C: toluene:cyclohexanol ratio. However, the interrelationships between the factors are more complex, as can be seen from the fact that not only the non-linear effects terms A^2^ and B^2^ are required to describe the influences on the response, but there is also a synergistic interaction (second order interaction term, 2FIA) between the factors BC, the ratio of the monomers and the composition of the porogen mixture. This means the effect of neither the monomer nor the porogen composition can be adequately discussed without considering the other factor. The statistical parameters for the single model terms and the overall model are collected in Table 5.

The model shown provides a very good indication of the complex effects of the process factors on the response pore size. It allows a very good correlation with the data R^2^ = 0.9900. Furthermore, a prediction of the pore size with an R^2^_predicted_ = 0.9452 is possible.

The effect of the synthesis parameters in coded form is given in Equation (8):

(8) $$\Phi_{50}/Å=200.92-167.93\;A+215.35\;B+127.15\;C+109.11\;\mathrm{BC}+98.38\;A^{2}+126.88\;B^{2}$$

The factor effect term B (GMA:EDMA) strongly influences the pore size of the particles, as both the linear and non-linear term show high positive values of factor effect coefficients. With an increase of GMA ratio the pore size increases, whereby the positive non-linear B^2^ term indicates the even stronger influence the higher the GMA ratio is. As far as the chemical composition is concerned this finding can be explained through decreasing cross-linking degree in the polymer structure, since the EDMA cross-linker content diminishes.

However, due to the pronounced 2FIA of GMA:EDMA and toluene:cyclohexanol ratio, the effect of the GMA content cannot be understood without at the same time considering the composition of the porogen mixture. Yet, the synergistic effect is less pronounced and the second smallest with a coefficient of +109.11.

**Figure 5:**
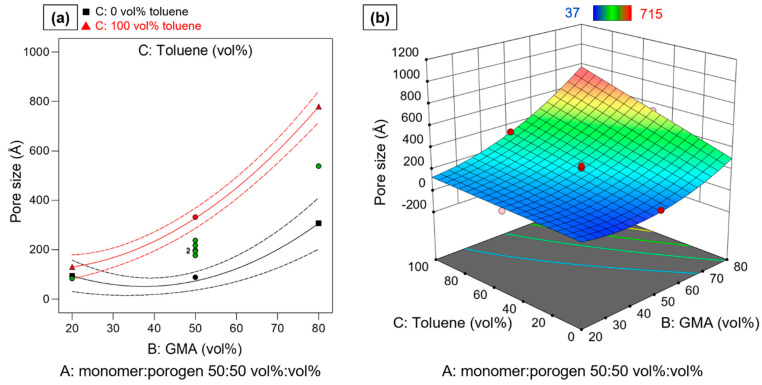
(**a**) Interaction plot of the synergistic interaction between GMA:EDMA*toluene:cyclohexanol at medium level of factor monomer:porogen ratio. Red triangles indicate measured pore size at high toluene content. Black squares indicate measure particle size at 0 vol% toluene (100 vol% cyclohexanol). Green circles indicate measured particle size at medium toluene level. Dashed lines indicate 95% confidential intervals. (**b**) Shows response surface of pore size in dependence of factors B and C at high level of A. Blue areas correspond to low pore sizes, red areas correspond to high pore sizes.

Figure 5a shows the interaction plot for the 2IA BC at a 50:50 vol% ratio for monomer:porogen. It can be seen that the higher the proportion of toluene in the porogen mixtures, the greater is the increase in pore size with increasing GMA content. This effect can be explained by the fact that with increasing GMA content toluene becomes less suitable as a solvent. As a result, phase separation starts earlier and earlier in relation to the gel point, which in turn leads to larger pores. A similar effect has already been shown for the pore volume.

The only factor with a negative effect on the pore size is “A”: an increasing ratio of monomer:porogen ratio results in smaller pore sizes. The positive effect of the non-linear term A^2^ shows a diminishing effect strength for higher monomer ratios, i.e., at higher monomer ratios the depleting effect on “shrinking” pore sizes is less pronounced. Therefore, according to the model, the highest pore sizes should be achieved with a low monomer:porogen ratio, a high amount of GMA or a low amount of EDMA crosslinker, and a high toluene ratio in the porogen mixture (see Figure 5b). However, it should be noted that this very extreme setting (−/+/+) also corresponds to the synthesis und reaction conditions Std7 which did not lead to any stable particles (compare Figure 2). This area of the experimental space represents a critical combination of process factor levels.

The largest pore size with stable particles was achieved in the experiment with reaction conditions Std8 (+/+/+) at a high monomer content. Even higher pore sizes for stable particles are expected up to a range of about 40–70 vol% monomer at high toluene levels. If lower amounts of toluene are used in the porogen mixture, even lower monomer ratios can lead to stable particles (Std3 (−/+/−) 631 Å).

### 3.6. Specific Surface Area

The specific surface area is an important characteristic for many applications that depend on surface interactions. Therefore, the specific surface area was determined as the third response value and calculated according to Equation (5).

The specific surface area is a function of the two previously discussed properties pore volume and pore size. Data analysis was again performed via an ANOVA which is given in Table 6. Of the linear terms, only B, the GMA:EDMA ratio is statistically significant by itself. The other linear terms are A: monomer:porogen and C: toluene:cyclohexanol were added to preserve model hierarchy, since they are involved in the significant interaction term AC. However, the non-linear effects A^2^ and B^2^ are highly statistically significant. Although the specific surface area is deducted from the pore volume and the respective pore size, a different combination of factor effects is statistically significant and required for model building. While the linear terms A and C were highly statistically significant for the other two parameters, these are only hierarchical in the model for the specific surface area. The interaction AC, on the other hand, was not relevant for any of the other responses. This shows that the relationship of the specific surface area and the process factors is highly complex and not predictable in a simple way. The relative impact of each process factor on the specific surface area can be seen in Equation (9) in terms of coded factors:

S/m^2^∙g^−1^ = 61.04 − 6.98 A − 64.34 B − 13.65 C + 52.74 AC − 28.16 A^2^ + 18.34 B^2^ (9)

The strongest effect on the specific surface area results from the negative coefficient for the B: GMA:EDMA ratio.

With increasing amount of GMA, the specific surface area is reduced. This general trend has been reported in literature [21,23,45]. However, through application of RSM the non-linearity of this effect (B^2^) was shown. The strength of the effect is leveled off with increasing GMA ratio as indicated by non-linear effect of B^2^. The terms A, C, A^2^ and C^2^ are strongly interrelated through the 2FIA term AC, which is the second largest effect, i.e., the system is strongly dominated by the synergistic behavior of two non-linear effects.

**Figure 6:**
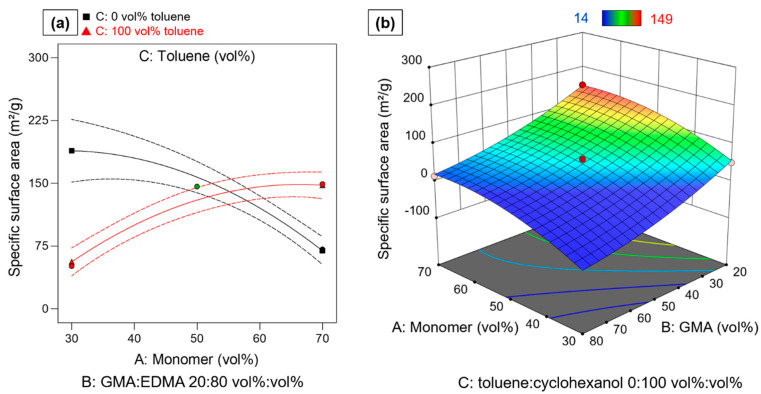
(**a**) Interaction plot of AC monomer:porogen×toluene:cyclohexanol at medium level of factor B for specific surface area. Red triangles indicate determined specific surface area at high toluene content. Black squares indicate determined specific surface area at 0 vol% toluene (100 vol% cyclohexanol). Green circles indicate determined specific surface area at medium toluene level. Dashed lines indicate 95% confidential intervals. (**b**) Shows response surface of specific surface area in dependence of factors A and B at low level of c. Blue areas correspond to low specific surface areas, red areas correspond to high specific surface areas.

The non-linear A^2^ term shows a maximum for the specific surface area, but since the factor A: monomer:porogen ratio is involved in an 2FIA with factor C (toluene:cyclohexanol ratio of the porogen mixture). This interaction results in a shift in maximum for the specific surface area, the maximum being strongly dependent of the level for A and C. This behavior is clearly visible in the interaction plot given in Figure 6a. For low toluene content a maximum specific surface area can be seen at a monomer content of approximately 35 vol%. With further increase in monomer ratio, the specific surface decreases. For high toluene content the opposite effect is visible. With increasing monomer ratio, the specific surface area increases until a maximum at around 60 vol% is obtained and then decreases again slightly. This effect is not influenced by the GMA:EDMA ratio of the monomers (Figure 6b). Again, this behavior can be explained by the different solubilities of monomers, PS seed and polymer particle in the porogens. These opposing effects could lead to the shift of maximal specific surface as a function of process factors levels.

This rather complex behavior can also be described by the model equation in terms of coded factor effect terms (Equation (9)). The model describes the data very well with R^2^ = 0.9748. It also shows a high predictive power with R^2^_predicted_ = 0.8678.

### 3.7. Model Validation

For validating the models for pore volume, pore size and specific surface area, four validation experiments were performed and particles were synthesized under reaction conditions that were not used to build the model. The corresponding factor level settings for the experiments are given Table 2. Table 7 lists the values for the response values that were predicted by the model and the actual values, as well as the residuals (the deviations from the model) for all validation points regarding pore volume, pore size and specific surface area.

It can be seen from Table 7, that all values predicted by the models are in good agreement with the experiments, except for the value for pore size of validation experiment Val4. This combination of process factor level settings is in an extreme area of the design space where small changes in process factors cause large changes in the response (high model sensitivity). All values for all of the three pore parameters pore volume, pore size and specific surface area were correctly predicted within the 95% predictive interval (PI) and show only small deviations (residuals) from the predicted values. Therefore, the models were confirmed to be correct and show a high predictive power. It should be particularly noted that the validation points were synthesized using a PS from a different batch to test the transferability of the models to other seed batches. As a result, the findings are also transferable to the other seed particle batch, which shows high model robustness.

## 4. Conclusions

In this work, the effects of the synthesis parameters (factors) monomer:porogen, the ratios of functional and crosslinking monomer GMA:EDMA and the composition of the porogen mixture of toluene:cyclohexanol on the particle properties pore volume, pore size and specific surface area of monodisperse porous poly-(glycidyl methacrylate-*co*-ethyleneglycole dimethacrylate) particles were systematically studied by applying response surface methodology or Design of Experiment.

The multivariate regression analysis (R^2^_predicted_ = 0.98) based on FTIR data of the porous particles showed that the proportion of functional epoxy groups in the porous p(GMA-*co*-EDMA) particles depends directly on the proportion of the functional monomer. This allows the targeted adjustment of the degree of functional groups contained in the platform particles, which is required for application-specific re-functionalization.

The effects of the synthesis factors on the pore volume, pore size and specific surface area parameters could all be described by robust and predictive models (R^2^_predicted_ 0.9012, 0.9452 and 0.8678, respectively). Non-linear effects of factors and synergistic interaction effects among factors were identified and quantified and were found to affect all response variables. This highlights the underlying complexity of seed swelling polymerization for the generation of porous monodisperse polymer particles.

The complexity of the interrelationships in this system could only be demonstrated by simultaneously considering numerous factors simultaneously by the RSM approach. The method of isolated consideration of individual synthesis parameters (one-factor-at-a-time approach), which has prevailed in the literature up to now, has not provided any information about interactions so far and cannot be expected to do.

Despite this complexity of the system, it was successfully achieved to obtain validated, robust models that allow prediction and tuning of particle properties such as pore volume, pore size, specific surface area with a certain amount of epoxy groups.

It can be assumed that other systems based on seed swelling polymerization, e.g., other monomers or porogen mixtures, exhibit similarly complicated behavior and are also subject to complex solubility phenomena. The approach described in this work was demonstrated to be suitable for evaluating such a system quickly and to deliver reliable and comprehensive information for enabling tailored adjustment of pore characteristics. Thus, RSM is a powerful tool for enabling the rational design of porous polymer particles.

## Figures and Tables

**Table 1:** Range of process factors level setting as used in the face-centered central composite experimental design (FCD).

Factor	Name	Low Setting (−1)	Center Point (0)	High Setting (+)
A	Monomer vol%	30	50	70
B	GMA vol%	20	50	80
C	Toluene vol%	0	50	100

**Table 2:** Factor level settings and the corresponding particle properties specific pore volume, pore size, specific surface area, particle size and dispersity d_90_/d_10_ listed according to Yates standard order (Std).

Std	Run	Factor Level Settings			Response Values				
		A	B	C					
		Monomer:Porogen Ratio	GMA:EDMA Ratio	Toluene:Cyclohexanol Ratio	Pore Volume *V_p_*	Pore Size $\Phi_{50}$	Specific Surface Area *S*	ParticleSize	Dispersity d_90_/d_10_
		/vol%	/vol%	/vol%	/mL∙g^−1^	/Å	/m^2^∙g^−1^	/µm	-
* 1	**2**	30	20	0	0.52	166	126	7.41	1.15
**2**	**5**	70	20	0	0.06	37	71	8.15	1.11
* 3	**3**	30	80	0	0.44	631	28	7.42	1.08
**4**	**7**	70	80	0	**n.a.**	**n.a.**	**n.a.**	7.74	1.20
**5**	**4**	30	20	100	0.55	426	51	8.82	1.16
**6**	**8**	70	20	100	0.18	49	148	9.41	1.06
**7**	**10**	30	80	100	**n.a.**	**n.a.**	**n.a.**	**n.a.**	**n.a.**
**8**	**9**	70	80	100	0.25	715	14	9.22	1.13
**9**	**1**	50	50	50	0.3	217	56	8.31	1.14
**10**	**6**	50	50	50	0.3	237	50	8.09	1.11
**11**	**11**	50	50	50	0.26	197	53	8.83	1.11
**12**	**13**	30	50	50	0.49	438	45	8.56	1.05
**13**	**19**	70	50	50	0.09	126	28	9.1	1.06
**14**	**18**	50	20	50	0.3	83	146	9.07	1.04
**15**	**14**	50	80	50	0.26	538	20	8.81	1.07
**16**	**15**	50	50	0	0.17	88	77	8.39	1.06
**17**	**17**	50	50	100	0.5	332	60	9.38	1.04
**18**	**16**	50	50	50	0.29	177	66	8.92	1.05
**19**	**12**	50	50	50	0.28	193	58	8.37	1.07
**Val1**	**20**	50	20	73	0.41	124	132	8.41	1.09
**Val2**	**21**	50	40	73	0.56	141	159	7.81	1.15
**Val3**	**22**	50	60	73	0.37	370	40	7.87	1.15
**Val4**	**23**	50	80	73	0.36	744	20	7.19	1.19

* marks model outliers.

**Table 3:** Actual versus predicted values of GMA:EDMA ratio/vol% and deviation.

Std	Actual GMA/vol% (GMA:EDMA)	Predicted GMA/vol% (GMA:EDMA)	Δ/vol%
Val1	20	19.8	−0.2
Val2	40	57.4	−2.6
Val3	60	76.6	−3.4
Val4	80	36.3	−3.7

**Table 4:** Analysis of variance (ANOVA) for the analysis of FCD design of pore volume.

Source	Sum of Squares	Df	Mean Square	F-Value	*p*-Value	
**Model**	0.2660	4	0.0665	77.64	<0.0001	significant
A-Monomer	0.1741	1	0.1741	203.24	<0.0001	
B-GMA	0.0026	1	0.0026	3.04	0.1118	
C-Toluene	0.0785	1	0.0785	91.59	<0.0001	
BC	0.0110	1	0.0110	12.88	0.0049	
**Residual**	0.0086	10	0.0009			
Lack of Fit	0.0074	6	0.0012	4.43	0.0856	not significant
Pure Error	0.0011	4	0.0003			
**Cor Total**	0.2746	14				

**Table 5:** Analysis of variance (ANOVA) for the analysis of FCD design of pore size.

Source	Sum of Squares	Df	Mean Square	F-Value	*p*-Value	
**Model**	5.341 × 10^5^	6	89,009.19	132.49	<0.0001	significant
A-Monomer	1.171 × 10^5^	1	1.171 × 10^5^	174.28	<0.0001	
B-GMA	1.563 × 10^5^	1	1.563 × 10^5^	232.68	<0.0001	
C-Toluene	54,489.80	1	54,489.80	81.11	<0.0001	
BC	21,969.58	1	21,969.58	32.70	0.0004	
A^2^	24,163.44	1	24,163.44	35.97	0.0003	
B^2^	40,191.80	1	40,191.80	59.82	<0.0001	
**Residual**	5374.59	8	671.82			
Lack of Fit	3217.79	4	804.45	1.49	0.3539	not significant
Pure Error	2156.80	4	539.20			
**Cor Total**	5.394×10^5^	14				

**Table 6:** Analysis of variance (ANOVA) for the analysis of FCD design of specific surface area.

Source	Sum of Squares	Df	Mean Square	F-Value	*p*-Value	
**Model**	20,322.72	6	3387.12	51.49	<0.0001	significant
A-Monomer	164.25	1	164.25	2.50	0.1527	
B-GMA	17,190.05	1	17,190.05	261.34	<0.0001	
C-Toluene	627.78	1	627.78	9.54	0.0149	
AC	5132.17	1	5132.17	78.02	<0.0001	
A^2^	1980.56	1	1980.56	30.11	0.0006	
B^2^	839.34	1	839.34	12.76	0.0073	
**Residual**	526.22	8	65.78			
Lack of Fit	379.02	4	94.75	2.57	0.1910	not significant
Pure Error	147.20	4	36.80			
**Cor Total**	20,848.93	14				

**Table 7:** Actual vs. predicted values (with low and high 95% prediction interval PI with alpha = 0.05) and corresponding residuals for the validation points Val1–4. Values exceeding the 95% PI range are written bold.

Std	Predicted/Actual/ Residual	Pore Volume/mL∙g^−1^	Pore Size/Å	Specific Surface Area/m^2^∙g
Val1	Predicted Value (±95% PI)	0.34 (0.27–0.42)	121 (48–194)	137 (115–160)
	Actual Value	0.41	124	132
	Residual	0.06	3	−5
Val2	Predicted Value (±95% PI)	0.35 (0.28–0.42)	185 (119–251)	78 (58–99)
	Actual Value	0.37	233	63
	Residual	0.02	48	−15
Val3	Predicted Value (±95% PI)	0.36 (0.29–0.43)	362 (297–427)	35 (15–56)
	Actual Value	0.37	370	40
	Residual	0.01	8	5
Val4	Predicted Value (±95% PI)	0.36 (0.29–0.44)	652 (576–728)	9 (0–33)
	Actual Value	0.36	**744**	20
	Residual	0.00	92	11

## Data Availability

Data are available upon request from the authors.

## Appendix A. Synthesis of Polystyrene (PS) Seed Particles

PS seed particles were synthesized using dispersion polymerization in alcoholic media. 20 mL styrene (99%) and 80 mL ethanol (96%) were added in a three-necked round-bottom flask (250 mL). After the addition of 1.0 g PVP k30 as stabilizer and 0.5 g BPO as initiator the suspension was sonicated for 10 min. The suspension was stirred with 120 rpm with a magnetic stirrer at room temperature for 30 min while purging with Argon. Thereafter the temperature was elevated to 70 °C for 24 h under reflux. The PS seed particles were centrifugated for 2 min at 7500 rpm and were washed three times with ethanol and three times with deionized water to rmove the reaction solution. The particles were freeze-dried under vacuum for 72 h.

## Appendix B. Explained Variance of Multivariate Calibration Model for Epoxy Functionalization

Appendix BFigure A1 shows the explained variance plot for the PLSR model building for epoxy functionalization in the validation, an explained variance with >90% is obtained using nine PLSR factors.
